# Non-Invasive Analysis of Bulk and Surface Remodeling of Non-Woven PLLA and Fiber-Sponge PLLA/Chitosan Scaffolds in Cell Culture Environment

**DOI:** 10.3390/molecules30234657

**Published:** 2025-12-04

**Authors:** Elena Khramtsova, Yulia Petronyuk, Christina Antipova, Roman Sharikov, Alexey Bogachenkov, Sergey Malakhov, Daria Bednik, Petr Dmitryakov, Timofei Grigoriev

**Affiliations:** 1 Emanuel Institute of Biochemical Physics, Russian Academy of Sciences, 119334 Moscow, Russia; alyonushk@gmail.com (E.K.);; 2 Department of Nanobiomaterials and Structures, National Research Center “Kurchatov Institute”, 123182 Moscow, Russia; 3 Moscow Center of Advanced Studies, 123592 Moscow, Russia

**Keywords:** non-woven matrix, developed surface, PLLA, chitosan, fibroblasts, scanning impulse acoustic microscopy, fiber-sponge structure, tissue-engineered constructs

## Abstract

The expanding application of three-dimensional matrices with complex surface topographies in regenerative medicine requires new methods to visualize and analyze the evolving elastic properties of tissue-engineered constructs (TECs) during maturation. In this study, scanning impulse acoustic microscopy (SIAM) was employed for the non-invasive investigation of non-woven matrices based on PLLA and its composites with chitosan. This technique was used to determine the speed of sound, integral attenuation, and spectral characteristics within the samples. The data obtained through acoustic microscopy were compared with the results from tensile testing, gel permeation chromatography, differential scanning calorimetry, scanning electron microscopy, and CCK-8 assays. The findings demonstrate that SIAM exhibits high sensitivity to alterations in the TEC’s composition, including the presence of functionalizing additives, embedded cells, and the subsequent processes of cell proliferation and extracellular matrix synthesis, as well as to changes in its geometric structure. Consequently, this methodology can be recommended as a powerful and non-destructive tool for the comprehensive monitoring of TECs throughout their in vitro maturation period.

## 1. Introduction

In regenerative medicine, the current trend focuses on the development of 3D matrices and tissue-engineered constructs (TECs). This represents a natural shift from 2D systems to more complex 3D structures that better mimic native cellular environments. When cells are cultured as monolayers on 2D surfaces (such as the bottom of Petri dish, film- or gel-like matrices, bulk devices), they develop polarity along the basal–apical axis. While this polarity is characteristic of certain bodily cell types, it is atypical for extracellular matrix (ECM)-forming cells like fibroblasts. As a result, 2D cultivation fails to maintain cell cultures in differentiated state and does not replicate their physiological condition. A whole range of biological processes (e.g., neovascularization) can only occur in a three-dimensional (3D) environment. Additionally, nutrient diffusion and cell migration within a 3D matrix differ significantly from those in traditional 2D in vitro culture systems [1]. In the human body, fibroblasts and the extracellular matrix maintain a complex dynamic relationship, with the cells exhibiting high sensitivity to changes in ECM signaling. This includes responses to deviations in mechanical properties or morphological features, such as surface microtopography [2]. The shift to 3D matrices also facilitates research into both onset and treatment of diseases, including cancer.

The above considerations demonstrate that 3D matrix fabrication methods should meet specific requirements to imitate the mechanical properties of the native tissues being replaced. Essentially, an artificial ECM should maintain its mechanical stability throughout the entire regeneration process until the natural ECM completely replaces it. Most soft tissues contain voluminous, porous loose ECM, which can be mimicked using non-woven and sponge-like polymer matrices. Experimental studies confirm that non-woven matrices promote cell adhesion and proliferation more effectively than solid polymer films, due to their high specific surface area and porosity. This enhancement is caused by more efficient nutrient diffusion and metabolic waste removal [3]. The local mechanical properties of the ECM, such as stiffness, act together with physicochemical cues to direct cell behavior, including migration, proliferation, and differentiation. From a cellular perspective, pore size dictates the geometry of cell adhesion, thereby influencing force transmission. For instance, pores larger than a cell promote growth along individual fibers, whereas smaller pores enable cells to bridge voids. This facilitates multipoint 3D adhesions that can enhance intercellular communication and ECM production [4]. Furthermore, matrices featuring high porosity, thin walls, and interconnected pores demonstrate long-term mechanical stability by eliminating autocatalytic reactions within the material [5].

Poly(L-lactide) (PLLA) is a biodegradable polymer widely used in biomedical applications. Its biocompatibility, biodegradability, and ability to be easily formed into 3D structures make PLLA an ideal candidate for TECs [6,7,8,9,10]. Fibroblasts effectively adhere to and proliferate on non-woven PLLA materials due to their porous structure, even without modifications. This cell behavior demonstrates the biomimetic properties of PLLA-based non-woven matrices for ECM restoration, including mechanical support, cell migration and nutrient diffusion [11]. However, the hydrophobicity of PLLA and its resulting poor cell adhesion require the development of modified PLLA-based materials [12]. The literature reports several surface modification techniques, including alkali or enzymatic treatment, plasma treatment and coating with hydrophilic polymers [13]. Modification with natural hydrophilic polymers improves matrix wetting and enhances cell adhesion [5]. Also, a fiber-sponge composite can imitate the structure of the native extracellular matrix, where the non-woven scaffold mimics the fibrous collagen network, and the chitosan sponge replicates the hydrated glycosaminoglycan components.

Chitosan, a natural wide-spread hydrophilic polymer, is particularly suitable for such modifications. It also has potential as an individual matrix material in tissue engineering. A possible drawback of chitosan, however, is its rapid degradation and subsequent loss of mechanical properties [14]. Cross-linking with agents like glutaraldehyde can address this limitation. By varying the chitosan content and degree of cross-linking, one can regulate the elastic properties of the composite in a wide range, while giving additional functional properties (antibacterial, adhesive, hemostatic, etc). Studies have demonstrated that chitosan-containing composite matrices stimulate fibroblast proliferation by improving their mechanical properties [15].

Despite the critical need for developing and characterizing 3D TECs, only a limited number of methods provide quantitative data and volumetric visualization of these structures. The most valuable approaches are non-invasive techniques that can be implemented under sterile conditions within a laminar flow cabinet. A key challenge arises from the matrices’ relatively large dimensions (up to 1 cm) and optical opacity. During cultivation, cells migrate throughout the matrix volume and orient themselves at various angles relative to the imaging lens. When stained with fluorescent viability markers, only cells in near-surface layers (approximately 100 μm deep) demonstrate adequate visibility. While confocal or two-photon microscopy can extend this imaging depth to about 500 μm, this remains insufficient for the volumetric imaging of 3D TECs applied in human regenerative medicine.

To detect morphological changes in matrices and monitor cell growth processes with a resolution of 0.1–20 μm, corresponding to the size of TEC structural components, one employs advanced visualization methods. Multiple studies have successfully utilized confocal laser microscopy, as well as integrated focused ion beam and scanning electron microscopy systems operating in both cryogenic [8,16] and environmental modes [17,18]. X-ray microtomography demonstrates particular utility when combined with heavy metal salt contrast agents [19]. Among the most promising approaches is scanning impulse acoustic microscopy (SIAM), which enables detailed examination of the internal opacical 3D microstructure of matrices and TECs under near-physiological conditions [20,21,22]. While the number of studies on acoustic microscopy for biomaterials is limited, existing research demonstrates its significant potential. For instance, SIAM has been shown to track cell and tissue development on acellular human cadaveric dermis [20]. Similarly, it can assess cell populations within hydrogel matrices [21]. Regarding non-woven matrices, preliminary studies have also described the PLLA non-woven behavior under tensile stress in an immersion environment [22]. The SIAM method has certain disadvantages, including a trade-off between resolution and imaging depth, as well as a low acoustic contrast between the immersion liquid and the composite elements. Despite these limitations, the micromechanical maps of scaffolds obtained with this method allow for the evaluation of matrix heterogeneity and provide a more comprehensive three-dimensional assessment.

In this study, we employed SIAM to assess the elastic properties of both polylactide-based non-woven polymer matrices and chitosan-containing fiber-sponge matrices. These evaluations were conducted both in their initial state and during cell incubation to provide insights into the evolution of surface-enhanced TECs. The SIAM approach enabled the characterization of these processes in relation to mechanical properties under near-physiological conditions.

Our findings establish a strong foundation for utilizing surface-enhanced non-woven matrices in developing advanced multicomponent and multilayer TECs, particularly those exhibiting nonlinear mechanical property changes during their evolution.

## 2. Results

### 2.1. Characterization of Obtained Matrices with Developed Surface

**Figure 1:**
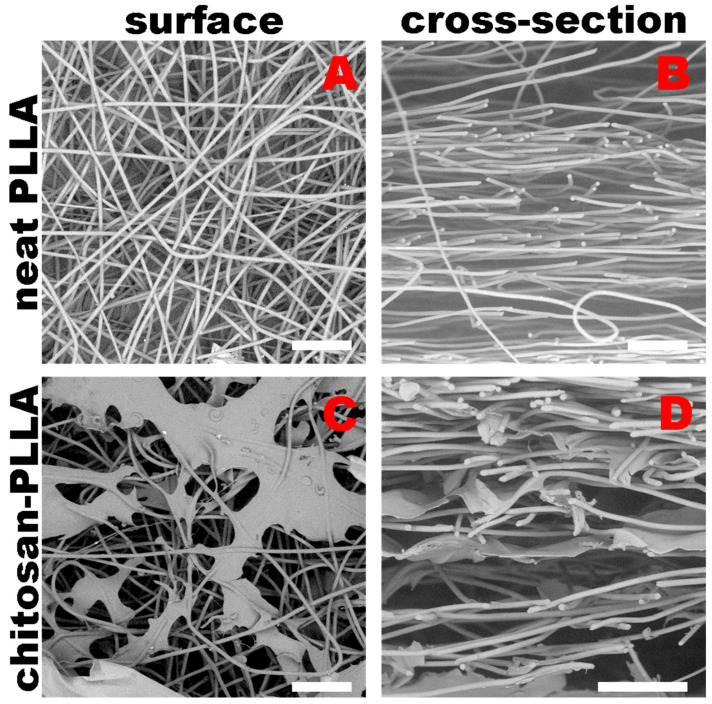
SEM images of non-woven materials: (**A**) Unmodified, surface; (**B**) Unmodified, cross-section; (**C**) With chitosan addition, surface; (**D**) With chitosan addition, cross-section. Bar 100 µm.

SEM images (Figure 1) were obtained for the intact non-woven matrices and composites immediately after preparation and sterilization. These images were used to evaluate the structural characteristics of the non-woven material, including its thickness (900 ± 20 μm), average fiber diameter (5.2 ± 1.1 μm), theoretically estimated specific surface area (0.625 m^2^/g), and fiber density (512 ± 63 fibers per mm^2^). The composite’s thickness was 480 ± 17 μm.

The fibers of the non-woven material exhibited surface porosity while remaining solid inside (Figure 1B). In the composites, chitosan film-like “bridges” were observed between the fibers, coating the pores at the points of contact. The SEM images confirmed that chitosan is not only present on the surface of the non-woven matrix but also penetrates into its bulk.

### 2.2. Gel Permeation Chromatography (GPC)

The changes in the molecular weight (M_W_) and polydispersity index (PDI) of the PLLA-based non-woven matrices were assessed using GPC. The results are summarized in Table 1. Unprocessed PLLA granules, which were used for the preparation of the electrospinning solution, served as the reference material, exhibiting an initial M_W_ of 155 kDa and a PDI of 2.6.

The electrospinning process, including the dissolution stage, was found to induce a substantial reduction in M_W_, exceeding 30%. At the same time, the additional preparation stage involving soaking in an acetic acid solution followed by lyophilization did not cause significant changes in the M_W_ of the modified samples.

Immersion of the samples in a culture medium further reduced the molecular weight by approximately 7% for the unmodified non-woven matrix, compared to only 3% for the composite material. This difference may be attributed to the presence of chitosan on the composite surface, which limits direct contact between the fibers and water. Subsequent incubation in the cell culture medium led to an additional 5% reduction in M_W_ for both materials.

It is hypothesized that during the initial days of exposure, chitosan undergoes swelling and then migrates into the culture medium, thereby facilitating liquid penetration into the polylactide fibers. As a result, the degradation rates of the modified and unmodified materials eventually converged.

### 2.3. Differential Scanning Calorimetry (DSC)

The thermophysical properties of the investigated PLLA-based matrices were examined using DSC. As presented in Table 2, the crystallinity of the PLLA-based non-woven matrices and the composites was determined. The reference PLLA granules exhibited a crystallinity of 47%.

### 2.4. Uniaxial Tension

**Figure 2:**
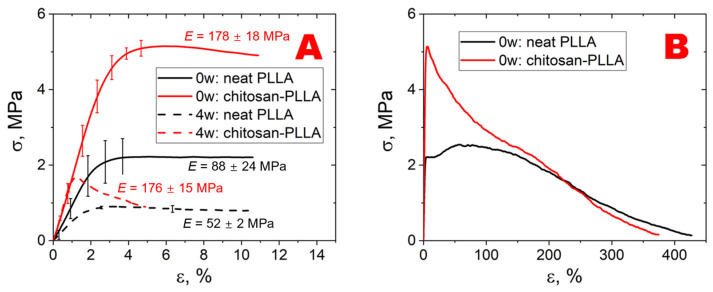
Uniaxial tensile stress–strain curves of the samples in the swollen state. (**A**) The linear elastic region (strain up to 15%); (**B**) The complete stress–strain curves.

Figure 2 demonstrates the stress–strain curves, obtained during uniaxial tensile testing of the studied samples within the isotropic X-Y plane.

### 2.5. CCK-8 Test Results

**Figure 3:**
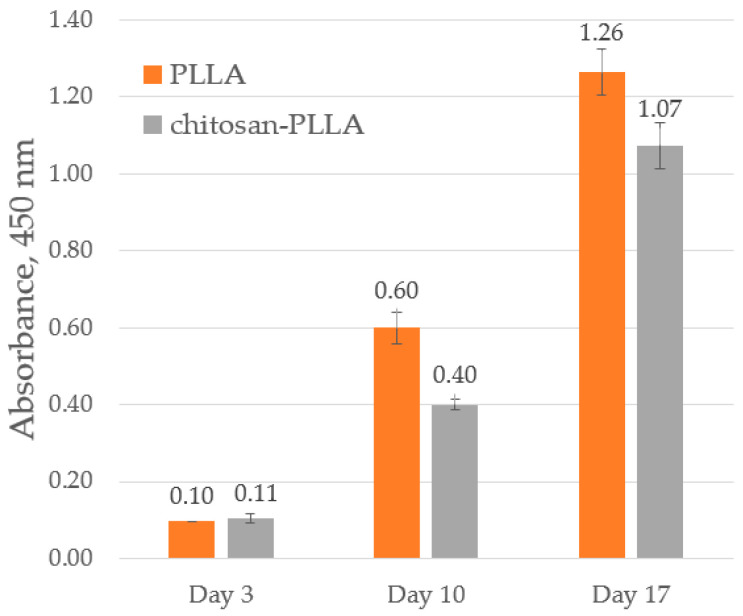
CCK-8 results of absorption at 450 nm of PLLA TECs and chitosan-PLLA TECs at different stages.

Figure 3 shows the absorption data measured at 450 nm.

The CCK-8 assay results demonstrate comparable cell numbers on both sample types at the initial post-seeding stage (Figure 3, day 3), consistent with the initial seeding concentrations. Subsequent cell growth was observed on both matrices, though it was moderately attenuated on the chitosan-modified composite relative to the neat non-woven matrix (Figure 3, day 10 and 17).

### 2.6. Scanning Impulse Acoustic Microscopy

#### 2.6.1. Ultrasound Visualization

**Figure 4:**
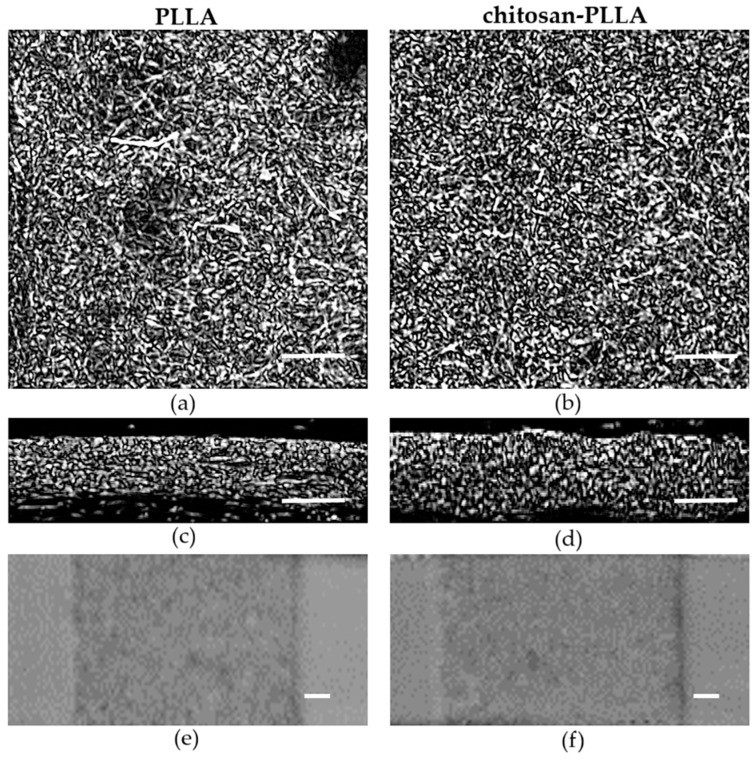
Ultrasound visualization: (**a**,**b**) Visualization of subsurface region (C-scans) of PLLA non-woven matrix and chitosan-PLLA composite; (**c**,**d**) Vertical cross-sectional image (B/D-scans) of PLLA non-woven matrix and chitosan-PLLA composite; (**e**,**f**) Integral microstructure on high-echogenic glass substrate (C-scans) of PLLA non-woven matrix and chitosan-PLLA composite. The scale bar is 500 µm.

Ultrasound images of the non-woven scaffolds were obtained from the central region of the samples after 3-day preconditioning period in DMEM (Figure 4).

Analysis of the samples revealed no significant differences in either surface or bulk microstructure following chitosan modification. Furthermore, integral imaging further demonstrated a high degree of homogeneity throughout the material volume (Figure 4e,f).

#### 2.6.2. Matrix Thickness

**Figure 5:**
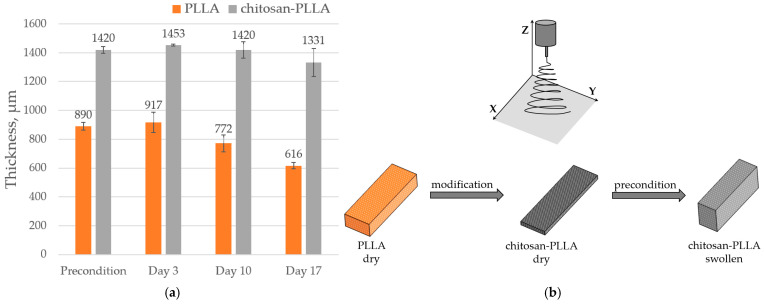
Changes in matrix thickness: (**a**) The thickness of non-woven PLLA and composite chitosan-PLLA matrices along the *Z*-axis, measured via acoustic microscopy, before cell seeding and throughout the subsequent cultivation period of the TECs. (**b**) A schematic representation of the alterations in the sample’s linear dimensions, illustrating the changes induced during composite fabrication and subsequent swelling, with reference to the orientation of the electrospinning nozzle along the *Z*-axis.

Acoustic microscopy performed in a DMEM environment enabled the determination of the samples’ effective thickness accessible to fibroblast migration and proliferation: 890 ± 26 μm for PLLA and 1420 ± 23 μm for the chitosan-PLLA composite (Figure 5a). While the non-woven PLLA scaffold remained dimensionally stable upon immersion, the composite samples exhibited a significant increase in thickness along the *Z*-axis due to chitosan swelling. This volumetric expansion was accompanied by a slight contraction of ≤10% in the linear dimensions along the X- and Y-axes (Figure 5b). Consequently, the matrix thickness along the *Z*-axis emerged as the primary parameter for monitoring structural changes. This metric also facilitated the observation of dynamic transformations during TEC maturation. Specifically, an initial thickness increase of approximately 30 μm was observed in both matrix types following cell seeding. As maturation progressed, the non-woven PLLA thinned by 274 μm, while the composite thinned by 89 μm.

#### 2.6.3. Speed of Sound (SoS)

**Figure 6:**
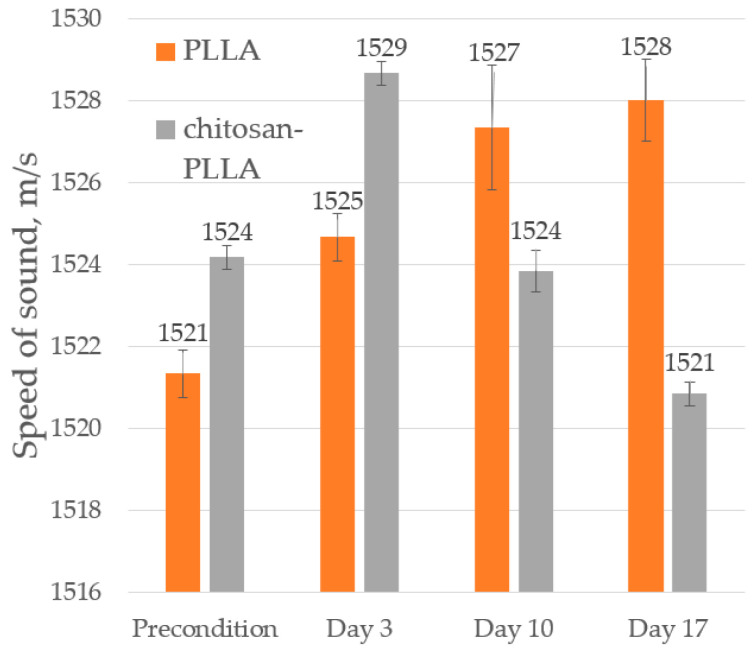
The speed of sound in non-woven PLLA and composite chitosan-PLLA materials before cell seeding and during TEC cultivation.

The speed of sound demonstrates greater sensitivity than acoustic imaging to the presence of both chemical modifiers and cellular infiltration within the matrix, as it is an integral characteristic that captures properties averaged across the entire sample volume (Figure 6).

Due to its high porosity of 92.5%, the SoS of the non-woven mat was close to that of DMEM: 1521 and 1509 m/s, respectively. In contrast, the composite mat exhibited a higher SoS of 1524 m/s (Figure 6), which correlates with its greater Young’s modulus (Figure 2). Following cell seeding on day 3, the SoS in both matrix types increased by approximately 4.5 m/s. This rise was consistent with the slightly higher SoS of cells compared to that of DMEM and corresponds to the equivalent initial cell number in both samples (Figure 3). From day 10 to day 17, a further increase in the SoS was observed in the non-woven matrix; however, these data exhibited considerable scatter, and the differences were not statistically significant.

Conversely, in the composite matrix over the same period, the SoS decreased to 1521 m/s, a change that occurred simultaneously with a reduction in the thickness of the TEC (Figure 5).

#### 2.6.4. Attenuation of Sound (AoS)

**Figure 7:**
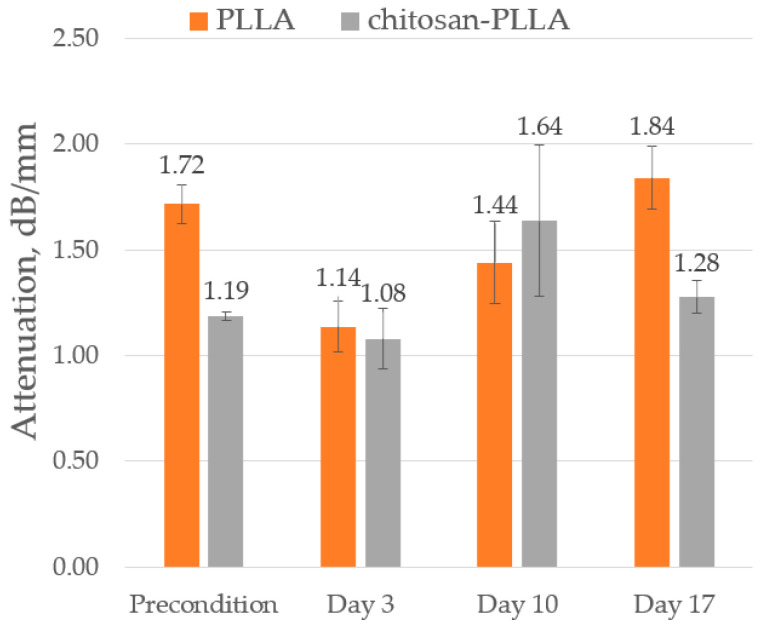
The sound attenuation diagrams of non-woven PLLA and composite chitosan-PLLA scaffolds, measured both before cell seeding and throughout the culture period of the TECs.

Prior to cell seeding, the non-woven matrix exhibited significantly higher AoS (1.72 dB/mm) compared to the composite matrix (1.2 dB/mm), as shown in Figure 7. Following cell seeding, the AoS converged for both matrices to a similar value of approximately 1.1 dB/mm. During the subsequent culture period from day 10 to 17, the AoS in the non-woven matrix increased to 1.84 dB/mm, a value that exceeded its initial pre-seeding level. In contrast, the composite matrix displayed a sharp increase in AoS to 1.64 dB/mm by day 10 (a measurement characterized by considerable data scatter) followed by a pronounced decrease to 1.28 dB/mm by day 17.

#### 2.6.5. Spectral Characteristics

**Figure 8:**
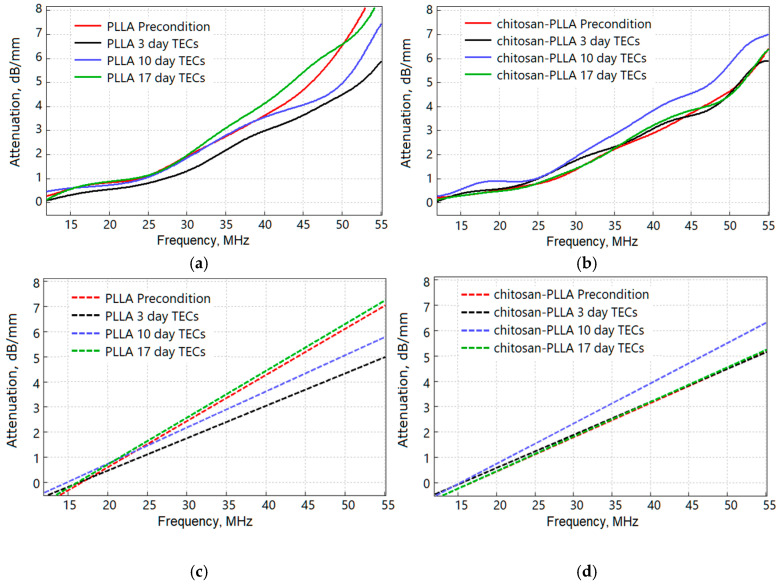
Frequency dependence of sound attenuation $R_{S}\left(f\right)$: (**a**) PLLA; (**b**) Chitosan-PLLA; and the linear approximation function $R_{S}^{\prime }\left(f\right)$: (**c**) PLLA; (**d**) Chitosan-PLLA.

The frequency dependence of the AoS, calculated using the spectral ratio $R_{S}\left(f\right)$, is shown in Figure 8a for the non-woven PLLA and in Figure 8b for chitosan-composite matrices. Linear fits to this spectral ratio $R_{S}^{\prime }\left(f\right)$ at various time points of cell culture are presented for the PLLA and composite matrices in Figure 8c and Figure 8d, respectively.

As illustrated in Table 3, the slope of these curves exhibits sensitivity to the inherent difference in scaffold composition, as observed in the preconditioned state, and remains responsive throughout all subsequent culture stages. Notably, the slope demonstrates a positive correlation with the overall integral attenuation previously shown in Figure 7; specifically, the non-woven scaffold without chitosan possesses a steeper slope than the composite. Following cell seeding, the slope for the non-woven scaffold increased to a value comparable to that of the pre-seeded composite scaffold. In contrast, seeding the composite scaffold with cells resulted in no significant change to this parameter. As maturation progressed, the slope for the non-woven PLLA TEC continued to increase. For the composite matrix, however, the slope intensified sharply by day 10, followed by a subsequent decrease.

**Figure 9:**
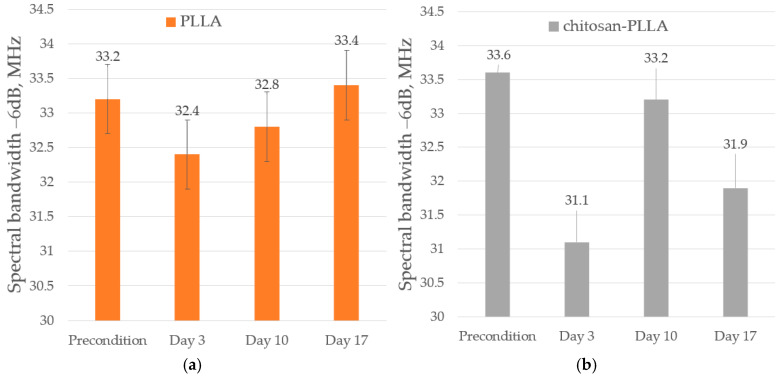
Spectrum bandwidth at −6 dB for: (**a**) Non-woven PLLA matrices and (**b**) Composite chitosan-PLLA matrices measured both before cell seeding and throughout the process of TEC formation.

Figure 9 shows the measured spectrum bandwidth (SBW) at −6 dB. A direct comparison of the samples on a single graph was limited by their differing thicknesses throughout the culture period. Analysis revealed a consistent narrowing of the SBW in both scaffold types following cell seeding. By day 10 of culture, the SBW had widened in both TEC types; however, a divergence in their spectral behavior emerged by day 17. Specifically, the PLLA-based TEC exhibited a continued widening of its SBW, whereas the composite TEC demonstrated a subsequent narrowing. This divergent behavior is consistent with the AoS data (Figure 7).

## 3. Discussion

The analysis of non-woven matrices with developed surface topographies presents several significant challenges for researchers. These include the difficulty of manipulating an easily deformable material, measuring the thickness of a subtle structure characterized by a loose surface and individually protruding fibers, and overcoming the low optical contrast inherent to a highly porous matrix with low polymer content. Furthermore, the fabrication of composites based on these non-woven matrices and the subsequent development of a TEC introduce additional complexity to the study of their dynamic behavior, as it requires a methodology that is both noninvasive and capable of examining the full sample volume. In response to these challenges, this work serves as a pilot study that utilizes acoustic microscopy to investigate the maturation of three-dimensional non-woven matrices and their composites into functional TECs.

### 3.1. Non-Woven and Composite Matrix in Precondition State

A comparative examination of the non-woven PLLA matrix and the chitosan-containing composite reveals that during fabrication in the dry state, the composite’s thickness was reduced to approximately 480 µm, a significant decrease from the 900 µm measured via SEM for the pure PLLA scaffold (Figure 1). This substantial reduction is attributed to the formation of chitosan bridges between the polymer fibers, which causes the adhesion of previously discrete fibers to one another [23]. Non-woven mats fabricated by electrospinning exhibit structural isotropy exclusively within the X-Y plane, a direct consequence of the manufacturing process which restricts the incorporation of vertically oriented fibers along the *Z*-axis. As a result, when infused with a low-viscosity chitosan solution, the chitosan predominantly binds fibers within individual horizontal layers. This leads to the formation of planar film-like structures in the X-Y plane rather than creating continuous vertical bridges. (Figure 1) [23].

**Figure 10:**
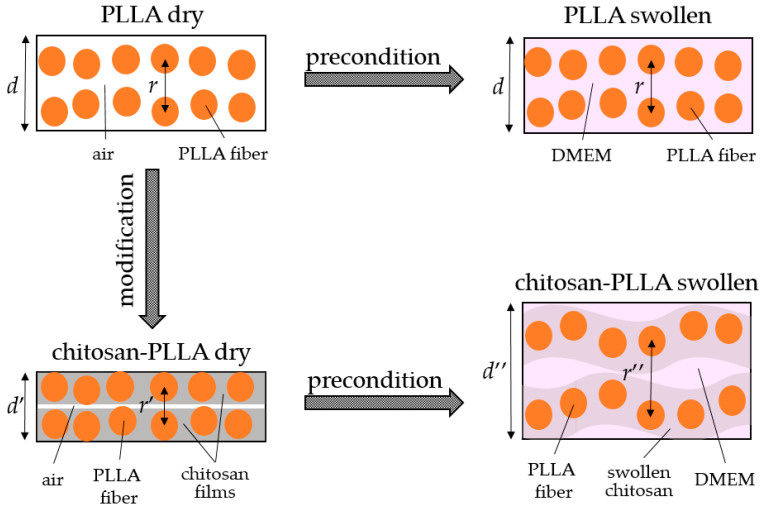
Schematic representation of the behavior of a non-woven matrix during modification and precondition: *d*—thickness and *r*—distance between layers in the Z direction of the non-woven matrix before manipulation; *d*′—reduced thickness and *r*′—reduced interlayer spacing in the Z direction of the composite matrix after modification with chitosan; *d*″—increased thickness and *r*″—increased interlayer spacing in the Z direction of the composite matrix after precondition.

Due to the highly hydrophilic nature of chitosan, acoustic microscopy revealed that during preconditioning in DMEM, the composite achieved a functional thickness of approximately 1400 μm under in vitro conditions. This significant expansion occurred with only minimal intrinsic swelling of the individual polymer fibers, which was measured at about 5% [22] (Figure 5a,b). Acoustic microscopy further demonstrated that the swelling of the composite mat is predominantly anisotropic, manifesting as a substantial 60% increase in thickness along the *Z*-axis, while the linear dimensions along the X- and Y-axes contract by ≤10%. This anisotropic behavior is attributable to both the specific architecture of the chitosan bridges and the inherent mechanical anisotropy of the non-woven matrix, which possesses a significantly lower Young’s modulus in the Z-direction compared to the X-Y plane (Figure 5b). During impregnation, the chitosan films swell and are forced to bend in the Z-direction, forcing the structure to expand vertically (Figure 10). A small number of bridges connect the layers of the non-woven matrix, preventing complete detachment of the layers, but the size of the swollen composite in the X- and Y-directions decreases.

Chitosan is not visible in the acoustic images (Figure 4), as its films within the composite are too thin, even in a swollen state, and exhibit a low acoustic impedance.

Despite the low degree of chitosan modification, its introduction into the system altered both the structure and the degree of swelling, while also significantly affecting the composite’s mechanical properties. The initial section of stress–strain curves (up to 10% strain) obtained from uniaxial tensile tests (in the X- and Y- directions) of the samples are presented in Figure 2. Mechanical tests results indicate that chitosan modification of non-woven fibrous matrices leads to a two-fold enhancement in Young’s modulus and changes the characteristic stress–strain behavior beyond the tensile strength (1–5% strain), where fiber rupture predominantly occurs (*p* < 0.05). For unmodified matrices at week 0, the curve exhibits a distinct plateau region, indicative of progressive rearrangement of intact fibers, followed by stress elevation due to fiber alignment along the loading axis, and subsequent gradual stress reduction resulting from individual fiber failures. In contrast, chitosan-modified composites display more abrupt stress reduction and accelerated fiber failure due to (a) structural constraints caused by chitosan inter-fiber bridging (as evidenced by SEM images) and (b) diminished inter-fiber interactions mediated by chitosan surface coatings [22,23]. These mechanical characteristics persist at week 4, though with reduced tensile strength values (*p* > 0.05). The considerable decrease in tensile strength observed can be attributed to residual stress relaxation during prolonged incubation in the culture medium. Notably, unlike the elastic properties of unmodified materials, the Young’s moduli of composite matrices remain consistent between initial measurements (week 0) and after 4-week incubation in culture medium (*p* < 0.05). This observation suggests that the slight reduction in molecular weight may be compensated by an increase in PLLA crystallinity. Furthermore, although chitosan predominantly migrates into the culture medium and desorbs from the material surface, persistent inter-fibrillar chitosan bridges within the matrix bulk likely contribute to the maintained elastic modulus over the 4-week period.

The DSC experimental results demonstrate that polymer processing and subsequent formation of the non-woven material induce amorphization of PLLA, irrespective of modification. As evidenced by the data in Table 2, brief exposure to aqueous or acidic media does not significantly change the crystallinity of PLLA. However, prolonged incubation in a culture medium at 37 °C results in a 10% increase in PLLA crystallinity, independent of the presence of chitosan in the system.

Alterations in the mechanical properties of the non-woven matrix resulting from its modification are also detected via acoustic microscopy. The SoS within the non-woven matrix is lower than in the composite; due to the low degree of modification, this difference is a 3 m/s (Figure 6, precondition). Conversely, the AoS in the composite is significantly lower than in the non-woven matrix (Figure 7, precondition). This discrepancy arises because ultrasound attenuation in such materials occurs through two principal mechanisms: absorption and scattering. As polymer materials are viscoelastic, a substantial portion of the attenuation occurs through absorption, which involves energy losses due to molecular motion and internal relaxation processes [24]. However, the structure of the non-woven matrix presents numerous interfaces between the PLLA and DMEM phases, which possess a significant acoustic impedance mismatch that results in a substantial contribution from scattering on individual fibers to the overall attenuation [25,26]. Chitosan modification smooths the composite surface (Figure 1 and Figure 10), enabling ultrasonic waves to penetrate the bulk material with reduced scattering. The acoustic impedance of the swollen chitosan modifier is intermediate between that of the DMEM and the polymer fibers, a characteristic that prevents an increase in AoS within the composite.

These observations are confirmed by the frequency dependence of AoS presented in Figure 8. The non-woven matrix in its preconditioned state (Figure 8a) displays a non-linear increase in attenuation in the high-frequency range (35–55 MHz), which is associated with scattering from individual fibers. This phenomenon is not observed in the composite material (Figure 8b), where the AoS level is 29% lower than in the pure non-woven matrix.

As the pronounced non-linearity in the attenuation profile is characteristic of the non-woven PLLA scaffold and diminishes significantly during the TEC formation process (days 3–17), subsequent analysis was performed using a linear approximation (Figure 8c,d). A direct comparison of the SBW between the non-woven and chitosan-composite scaffolds was not conducted due to substantial differences in their sample thicknesses.

### 3.2. TECs Formation Day 3

CCK-8 analysis conducted on day 3 following cell seeding revealed a comparable number of viable cells on both matrix types (Figure 3, day 3). In [11] it was established that immersion of a non-woven PLA matrix in complete DMEM, in the absence of cells, resulted in the deposition of a surface film on the fibers within 24 h, presumably composed of proteins from the medium. This suggests that even a synthetic polymer becomes more conducive to cell attachment following preconditioning prior to seeding. Modification with chitosan, a hydrophilic, biocompatible, and positively charged natural polymer, is anticipated to further enhance protein adsorption from the DMEM. This mechanism establishes a more natural interface between the polymer and the cells, facilitating adhesion via integrin contacts and subsequent initiation of de novo ECM synthesis.

Direct visualization of the cells themselves, either on the surface or within the bulk of the matrix, was not feasible under these experimental conditions using acoustic microscopy. This limitation arises because the acoustic impedance of the cells is similar to that of the DMEM, and their positioning at various angles on the loose fibrous surface, where their size becomes comparable to the wavelength in the immersion, precludes clear imaging. Nevertheless, acoustic microscopy detected an increase in the thickness of both matrix types by day 3 (Figure 5, day 3).

A concurrent increase in SoS of approximately 4 m/s was observed in both cases (Figure 6, day 3), confirming the presence of cells within the systems.

Furthermore, a decrease in AoS was recorded for the non-woven matrix upon cell seeding. This attenuation reduction is attributed to cells adhering preferentially at fiber intersections, thereby smoothing the surface topography. While the majority of cells initially reside on the matrix surface, their migration into the bulk occurs through fiber displacement, a process that does not require direct fiber destruction [4]. Simultaneously, the pores of the composite matrix are partially occluded by chitosan. Within this structure, the fibers are embedded within a chitosan gel, which restricts significant fiber displacement by the cells. This confinement limits initial cell penetration and promotes an attachment more closely aligned with two-dimensional (2D) culture. Consequently, the AoS of the composite exhibits minimal change at this stage (Figure 7, day 3).

The alteration in the slope of the frequency-dependent attenuation correlates with AoS data; cell seeding reduces the slope for the non-woven matrix, whereas it remains virtually unchanged for the composite (Figure 8, day 3). However, cell seeding exerts a pronounced effect on the SBW, which narrows in both cases but to a greater extent in the composite (Figure 9, day 3). This suggests that the surface of the composite matrix is uniformly smoothed by the combined presence of the chitosan modifier and adhered cells, whereas the surface of the non-woven matrix is only partially covered and smoothed by cellular attachment.

### 3.3. TEC Formation Day 10

A previous study [27] demonstrated that under static cultivation conditions, active cellular colonization throughout the volume of non-woven PLLA matrices with comparable fiber diameter and porosity commences by the seventh day. In our case, a significant divergence in cell mass growth was observed via CCK-8 assay between the non-woven and composite matrices by day 10 of culture (Figure 3, day 10). This phenomenon may be attributed to a combination of factors. Firstly, the altered microarchitecture and reduced porosity of the composite scaffolds likely obstruct cellular infiltration into the bulk, thereby confining proliferation predominantly to the superficial layer. Secondly, the distinct local elastic modulus (Figure 2) and surface composition of the fiber-sponge material provide a different mechanical and biochemical microenvironment. This is sensed by the cells, potentially leading to flattened, more two-dimensional growth characterized by altered focal adhesion and differential synthesis of ECM components in different proportions compared to the pure non-woven matrix [28].

A reduction in matrix thickness was observed by day 10 (Figure 5a). For the non-woven matrix, this thinning is attributed to cellular migration into the bulk, proliferation, and the consequent binding of PLLA fibers by cells, coupled with the initiation of volumetric ECM synthesis. In the composite matrix, the thickness decreased to its preconditioned level but exhibited a greater spread of values, indicating the emergence of regions with non-uniform structure.

The SoS increased within the non-woven matrix (Figure 6, day 10), a change that correlates with the rising cell population and the potential onset of ECM synthesis. Considering the concurrent decrease in matrix thickness and the preservation of PLLA fibers at this stage (Table 1 and Table 2), the packing density of the TEC appears to increase. In contrast, SoS within the composite matrix decreased. Given the confirmed growth of the cell culture (Figure 3, day 10) and the stability of the PLLA fibers, it may signify the initial stages of chitosan degradation in the cellular environment. That hypothesis indirectly supported by the observed decrease in thickness.

The AoS increased by day 10 in both scaffold types, although the composite demonstrated a significantly higher value with greater data dispersion (Figure 7, day 10). The spectral slope (Figure 8c) and the SBW (Figure 9) also increased, exhibiting a more gradual rise in the non-woven TEC and a sharp increase in the composite. These divergent acoustic signatures correlate with the incipient degradation of chitosan in the composite and the formation of distinct ECM types within the two different scaffold environments.

### 3.4. TECs Formation Day 17

By day 17, the rate of cell mass growth in the composite scaffolds remained lower than in the non-woven ones (Figure 3, day 17), although active proliferation persisted. This sustained activity is likely facilitated by the emergence of new migration pathways as the chitosan, which initially impedes infiltration, undergoes breakdown.

The thickness of both matrix types continued to decrease (Figure 5, day 17). In the non-woven matrix, this is attributed to an increasing number of fibers being incorporated into the cellular mass and enveloped by the synthesized ECM. The composite matrix experiences these same processes, compounded by the ongoing degradation of chitosan and the consequent diminishment of its structural contribution. However, the composite’s thickness decreased to a lesser extent than that of the non-woven matrices, as the residual chitosan gel still provides mechanical resistance to contraction by cells.

The SoS increased in the non-woven PLLA scaffold, consistent with rising TEC density from continued cell proliferation and ECM deposition. Conversely, the SoS decreased in the composite, reflecting the combined effects of the aforementioned processes (Figure 6, day 17).

The AoS in the PLLA scaffold continued to increase, resulting from the growth and structural maturation of the native ECM (Figure 7, day 17). In the chitosan-PLLA scaffold, however, attenuation decreased, a consequence of two factors: the continued degradation of chitosan, which reduces its mechanical influence, and the synthesis of a remodeled ECM.

The observed decrease in both the spectral slope (Figure 8c, day 17) and the SBW (Figure 9, day 17) for the fiber-sponge composite, relative to the non-woven matrix, further indicates alterations in the composition and structure of the nascent ECM. This acoustic signature reflects the ongoing remodeling of the composite’s ECM as the mechanical properties of the scaffold dynamically changed. In contrast, the more stable mechanical microenvironment of the non-woven matrix resulted in smoother, more gradual changes in its acoustic characteristics throughout the TEC formation process.

To consolidate the understanding of the dynamic processes occurring during TEC maturation, a summary of the findings is provided in Table 4.

This study demonstrates that SIAM exhibits high sensitivity to dynamic remodeling of the component composition of non-woven matrices with the developed surface topography during the evolution of TECs. Furthermore, this technique enables the non-invasive evaluation of spatial variations in elastic properties and sample geometry under standard in vitro culture conditions. Analysis of spectral characteristics in relation to AoS revealed distinct differences in the predominant attenuation mechanisms [25,26], which were systematically associated with specific stages of TEC maturation.

## 4. Materials and Methods

### 4.1. Matrix Preparation

#### 4.1.1. Non-Woven Fibrous Matrix Preparation

Non-woven materials were fabricated via electrospinning from a 9 wt% solution of polylactic acid (PLLA) in a solvent mixture of chloroform and ethanol (90:10 by volume) [29]. The electrospinning process was carried out on a setup developed at the Kurchatov Institute Research Center under ambient temperature and the following processing parameters: an applied voltage of 19 ± 1 kV, a capillary-to-collector distance of 30 ± 1 cm. A dual-capillary system was employed for polymer solution delivery, with a volumetric flow rate of 10 mL/h per capillary. The PLLA solution was supplied using a syringe pump (DS-08, Visma-Planar, Minsk, Belarus). High voltage was applied to the capillaries via a Spellman SL130PN30 power supply (Spellman High Voltage Electronics Corporation, NY, USA). To prevent solvent evaporation at the capillary tips, a solvent vapor (chloroform) stream was applied at 28 °C.

A slowly rotating cylindrical drum collector was used as the electrode, operating at a rotational speed of 30–60 rpm with additional axial movement to ensure uniform fiber deposition. The working width and diameter of the drum were 30 cm and 14 cm, respectively. All experimental samples were subjected to vacuum drying (1 mbar) for at least 24 h at room temperature to remove residual solvent before further characterization.

#### 4.1.2. Fiber-Sponge Composite Preparation

The non-woven polylactide-based materials were modified using a 0.5 wt% aqueous chitosan ChitoClear HQG800 (Primex, Siglufjörður, Iceland) M_w_ = 600 kDa, degree of deacetylation of 60% solution in acetic acid [29]. To enhance hydrophilicity, the materials were first wetted with ethanol, followed by solvent exchange with water, and then immersed in the chitosan solution. After 24 h of impregnation, the samples were removed, blotted to remove excess solution, and frozen at –22 °C. The frozen specimens were then freeze-dried for 48 h in a Alpha 2-4LSC lyophilizer (Martin Christ, Osterode am Harz, Germany). The composite materials were crosslinked by exposure to glutaraldehyde vapor (25 wt% aqueous solution) for 30 min.

The chitosan content in the modified non-woven matrix was determined gravimetrically, yielding a loading efficiency of 7.9 ± 2.6%.

### 4.2. Matrix Characterization

#### 4.2.1. Scanning Electron Microscopy (SEM)

The SEM image of the PLLA-based matrices and the composites was obtained using scanning electron microscope Phenom XL (Thermo Scientific, Waltham, MA, USA) with a backscattered electron detector at an accelerating voltage of 5 kV and a pressure of 60 Pa without a conductive coating.

#### 4.2.2. Microstructure Characterization

The structural characteristics of the PLLA-based matrices and the composites were investigated through quantitative analysis of SEM images using Scope Photo 3.1 (ScopeTek, Hangzhou, China) image analysis software. Fiber diameter distribution was determined by measuring a minimum of 100 individual fibers per sample to ensure statistical significance. Cross-sectional analysis of vertically oriented samples enabled simultaneous determination of material thickness, individual layer dimensions, and fiber density per unit area.

To preserve native fiber architecture and prevent structural deformation during preparation, the samples were cryogenically sectioned in liquid nitrogen using a precision blade. The number of fibers was counted within defined microscopic fields of known dimensions, with subsequent normalization to fiber count per square millimeter. The theoretical specific surface area was evaluated from the mean average fiber diameter ($a_{avr}$) and the bulk density of PLLA (ρ = 1.24 g/cm^3^) using formula:

(1) $$S=\frac{4}{\rho \cdot a_{avr}},$$

The results were processed using Origin Pro 9.5.1.195 (OriginLab, Northampton, MA, USA) software.

### 4.3. TEC Formation and Hydrolysis

#### 4.3.1. Preconditioning of Matrix

Standardized samples (5 mm × 7 mm for cell seeding and 5 mm × 20 mm for GPC, DSC and mechanical tests) were punched out from both the composite and unmodified non-woven matrices. The prepared specimens were placed in SteriT sterilization bags (LLC “NPF ‘Vinar’”, Moscow, Russia) and loaded into a sterilization unit with a ^60^Co γ-radiation source (NRC Kurchatov Institute, Moscow, Russia). The distance and irradiation time were calculated to achieve a dose rate of 1 Gy/s, with an absorbed dose of 15 kGy.

To enhance surface hydrophilicity, the samples were immersed in 96% ethanol for 60 min under laminar flow cabinet conditions, followed by three sequential 30-min washes in phosphate-buffered saline (PBS) to remove residual alcohol. The conditioned matrices were subsequently incubated for 24 h in complete Dulbecco’s Modified Eagle Medium (DMEM) supplemented with glutamine, 4.5 g/L glucose (PanEco, Moscow, Russia), 10% defined fetal bovine serum (Global Kang Technology, Qinhuangdao, China), and an antibiotic-antimycotic solution containing penicillin, streptomycin, and amphotericin B (HiMedia, Thane, India). This equilibration step was performed in untreated 48-well culture plates (ServiceBio, Wuhan, China) under standard culture conditions (37 °C, 5% CO_2_) to ensure complete non-woven matrix hydration and chitosan swelling.

#### 4.3.2. Cell Seeding

Primary rat dermal fibroblasts at passage 5 were employed as the cellular component for a TEC fabrication. Based on established protocols for surfaces with developed topography [30], a seeding density of 100,000 cells/cm^2^ was utilized, corresponding to 35,000 cells per matrix sample given a seeding area on the upper side of 5 mm × 7 mm. For each experimental group, nine TEC samples were prepared, with three replicates allocated for each designated time point. The culture medium in all the TECs was refreshed every 48 h, and cellular proliferation was quantitatively assessed on days 3, 10, and 17 using the CCK-8 assay.

#### 4.3.3. CCK-8

Fibroblast proliferation on the non-woven PLLA-based scaffolds and the composites was evaluated using the Cell Counting Kit-8 (CCK-8) (Vazyme, Nanjing, China). This test utilizes WST-8 [2-(2-methoxy-4-nitrophenyl)-3-(4-nitrophenyl)-5-(2,4-disulfophenyl)-2H-tetrazolium, monosodium salt], a highly sensitive tetrazolium salt analogous to MTT that is reduced to water-soluble formazan by mitochondrial dehydrogenases in viable cells. The resultant formazan concentration exhibits direct proportionality to the number of metabolically active cells, enabling quantitative proliferation assessment.

Compared to conventional MTT test, the CCK-8 method offers distinct advantages: (a) reduced test duration, and (b) preservation of cell viability post-testing. Following medium replacement to remove CCK-8 reagents, cells remain viable for subsequent cultivation or additional analyses, thereby enhancing experimental reproducibility and data reliability.

For test implementation, 50 μL of CCK-8 solution was added to each well containing 500 μL of culture medium, followed by incubation for 2 h at standard culture conditions. Absorbance measurements were performed at 450 nm using a multimodal microplate reader Varioskan LUX (Thermo Scientific, Waltham, MA, USA). Control wells containing complete culture medium and CCK-8 reagent without cells were included to establish baseline absorbance values.

#### 4.3.4. Hydrolysis

To monitor hydrolytic degradation of PLLA matrices, rectangular specimens measuring 5 mm × 20 mm were employed. These acellular samples underwent hydrolysis under identical conditions and culture medium as the TECs, with synchronous medium changes performed in parallel with cell-containing samples. Degradation kinetics were assessed at two time points: 0 weeks and following 4 weeks of incubation.

### 4.4. Gel Permeation Chromatography

Molecular weight characteristics were determined by gel permeation chromatography (GPC) using a Knauer system equipped with a degasser, a Smartline Pump 1000 and a refractive index detector Smartline RI Detector 2300 (Berlin, Germany), a JetStream thermostat (DURATEC Analysentechnik GmbH, Hockenheim, Germany), and two Agilent PLgel Mixed-C columns 300 × 7.5 mm (Agilent Technologies Inc., Santa Clara, CA, USA) connected in series. Tetrahydrofuran (THF) was used as the mobile phase. The experiments were conducted at 40 °C with a flow rate of 1 mL/min. Calibration was performed using polystyrene standards covering a molecular weight range of 162 to 1290 kDa. Data were processed with ClarityChrom software 9.1 GPC module (Knauer, Berlin, Germany).

### 4.5. Differential Scanning Calorimetry

The thermophysical properties were characterized using a PerkinElmer DSC8500 (PerkinElmer, Inc., Springfield, IL, USA) compensation-type differential scanning calorimeter. Measurements were conducted over a temperature range of 0 to 220 °C with a heating rate of 20 °C/min, achieving a temperature accuracy of ±0.1 °C. All experiments were performed under a continuous nitrogen atmosphere with a flow rate of 20 mL/min. Samples were prepared in standard non-hermetic aluminum crucibles (40 μL capacity) sealed with press-fit lids. Precise sample weighing was performed using a Sartorius CPA225D (Sartorius AG, Gottingen, Germany) analytical balance with an accuracy of ±0.01 mg. The degree of crystallinity of the PLLA was calculated using the formula:

(2) $$\alpha =\frac{∆H_{cryst}{-∆H}_{melt}}{\omega \cdot ∆H_{melt}^{0}},$$

where ${\Delta H}_{cryst}$ is the enthalpy of crystallization, ${\Delta H}_{melt}$ is the enthalpy of melting of PLLA in the studied specimen, ω is the mass fraction of PLLA, and ${\Delta H}_{melt}^{0}$ = 93 J/g [31].

### 4.6. Uniaxial Tension

The mechanical properties of fibrous non-woven matrices were evaluated using rectangular specimens with a 5 × 10 mm working area. Uniaxial tensile testing was performed on an Instron 5965 universal testing machine (Instron, Norwood, MA, USA) at a constant speed of 25 mm/min. The elastomeric properties of the fibrous materials were characterized by Young’s modulus, tensile strength, and elongation at break. For statistical reliability, three replicates were tested for each sample type.

Due to the inherent difficulty in measuring the actual cross-sectional area of fibrous non-woven materials, the elastic properties were calculated based on the nominal cross-sectional area. This was determined using the sample mass and polymer density, with stress (σ) calculated as:

(3) $$\sigma =\frac{F}{S_{r}}=\frac{F\cdot l\cdot \rho }{m},$$

where F—applied force (N), $S_{r}$—nominal cross-sectional area (mm^2^), l—gauge length of the specimen (mm), ρ—polymer density (g/cm^3^), m—sample mass (g).

Mechanical testing data were analyzed using two-way ANOVA with material type and hydrolysis time as factors, followed by Tukey’s post hoc test for multiple comparisons. Statistical significance was set at *p* < 0.05. Data are presented as mean ± standard deviation (*n* = 5).

### 4.7. Scanning Impulse Acoustic Microscopy

In our studies, Scanning Impulse Acoustic Microscope SIAM-2017 (Emanuel Institute of Biochemical Physics, Russian Academy of Sciences, Moscow, Russia), was employed. Comprehensive descriptions of the used quantitative methodologies and imaging modalities are available in prior publications [22,32].

#### 4.7.1. Image Acquisition

Ultrasonic imaging was performed using a 50 MHz acoustic lens with a 30-degree angular aperture (Emanuel Institute of Biochemical Physics, Russian Academy of Sciences, Moscow, Russia). Near-surface regions of the samples were characterized in C-scan mode, while the central volume was assessed using B/D-scan mode with dynamic depth focusing. This configuration yielded a lateral resolution of 36 ± 18 μm.

#### 4.7.2. Quantitative Measurements

In this experiment, a focused ultrasonic pulse was generated by a 50 MHz lens possessing an 11.2-degree angular aperture (Emanuel Institute of Biochemical Physics, Russian Academy of Sciences, Moscow, Russia) within an immersion medium of complete DMEM. This setup produced a focal beam with a waist diameter of 135 μm and a focal length of 4.5 mm. The sample, with a thickness of up to 1.5 mm, was positioned entirely within this focal waist region upon a highly echogenic glass substrate with known acoustic properties [33,34]. The ultrasonic beam propagated through the sample, reflected from the substrate interface, and was subsequently received by the acoustic lens. For the measurements, signals were recorded both with and without the sample present, maintaining identical environmental conditions at 23 °C and 60% relative humidity. The excitation parameters for the ultrasonic probe pulse remained consistent throughout all measurements, utilizing short pulses of 20 ns duration. Signal acquisition was performed within a frequency range of 10–70 MHz, with a voltage of 20 V applied to the piezoelectric transducer of the lens. Following propagation, the signals were digitized at a sampling frequency of 500 MHz.

The speed of sound was determined using the following an equation [35]:

(4) $${SoS}_{S}=\frac{t_{DMEM}}{t_{S}}\cdot {SoS}_{DMEM}$$

where ${SoS}_{S}$—the speed of sound in the sample, ${SoS}_{DMEM}$—the speed of sound in the immersion medium (complete DMEM), $t_{DMEM}$ and $t_{S}$—the time of echo pulse propagation to the glass substrate and back in the absence and presence of a sample, respectively. In our conditions, ${SoS}_{DMEM}$ was determined in advance 1509 m/s and was taken into account in all subsequent measurements. The delay time was consistently measured at the point of the maximum positive amplitude in the signal. The systematic measurement error for the delay time was ±2 ns, corresponding to a speed measurement error of ±0.5 m/s.

Attenuation was calculated using the following equation [34,36]:

(5) $${AoS}_{S}=\left(20lg\frac{A_{DMEM}}{A_{S}}\right)\frac{1}{2d}$$

where ${AoS}_{S}$—coefficient of sound attenuation in the sample [dB/mm], $A_{DMEM}$—the amplitude of the signal reflected from the substrate when passing through immersion, $A_{S}$—the amplitude of the echo signal from the substrate when ultrasound passes through the sample, d—the sample thickness. The amplitudes of the echo signals were consistently measured at the fixed focal position on the substrate surface, and consisted of the maximum and minimum of the recorded echo signal.

When measuring the SoS and the AoS, in both cases the thickness of the sample is involved in the calculations (Equations (4) and (5)). For thin non-woven samples, determining the thickness is challenging due to changes during swelling and the presence of protruding fibers, which produce a loose, non-uniform surface topography (Figure 4c,d). To mitigate these effects, we used equilibrium-swollen samples fabricated to a specific geometry (see Section 4), which ensured a highly uniform distribution of fiber thicknesses and packing density within the deposition plane [22]. For the attenuation measurements, the ultrasonic beam was propagated through the sample thickness, with the entire volume of polymer fibers within the focal region contributing cumulatively to the recorded acoustic signal.

More comprehensive information on broadband ultrasonic attenuation was obtained by analyzing its frequency dependence using a standard Fourier transform. The data were prepared for spectral analysis by extracting a 200-ns segment from the original echo signals, centered on the positive peak. This segment was zero-padded to a total length of 4096 points for the subsequent fast Fourier transform. As the signal amplitudes near the boundaries of the original segment were negligible, no windowing function was applied. The spectral ratio was then calculated using an equation below (specifically by subtracting the curves presented on a logarithmic scale) [33,36,37,38,39,40]:

(6) $$R_{S}\left(f\right)=\left(20lg\frac{F_{DMEM}\left(f\right)}{F_{S}\left(f\right)}\right)\cdot \frac{1}{2d}$$

where $F_{DMEM}$—DMEM spectrum, a $F_{S}$—spectrum of the signal passed through the sample, d—the sample thickness. For the comparative analysis of samples during the growth of the TECs in both the composite and pure PLLA matrix, a function $R_{S}^{\prime }\left(f\right)$ representing a linear approximation of $R_{S}\left(f\right)$ within the 15–55 MHz range was employed.

Furthermore, a comparative analysis of samples, both with and without the modifier, was conducted at various incubation time points with cells by measuring the spectral bandwidth (SBW) of the echo pulses at a −6 dB level. The uncertainties in determining the frequency parameters were governed by the sampling frequency and the number of samples, amounting to ±0.1 MHz in this instance.

### 4.8. Statistical Analysis

All experiments were conducted in five independent experiments and five measurements per sample in each experiment. Results shown as mean ± standard deviation. Differences were statistically significant at *p* ≤ 0.05.

## 5. Conclusions

Previous studies have investigated scaffold rigidity and its dynamically evolving mechanical properties using a variety of methodologies, ranging from nanoindentation and mechanical loading to atomic force microscopy. However, these methods become less applicable with the increasing complexity of matrices dimensionality and physiologically relevant culture conditions. Moreover, the absence of a standardized system for evaluating matrices and tissue-engineered constructs in laboratory practice complicates the investigation of the subtle mechanisms governing tissue formation on synthetic scaffolds in the context of their viscoelastic properties.

This study demonstrates that the combined application of quantitative ultrasound techniques and the CCK-8 viability assay provides a highly effective means of characterizing all stages of TEC maturation. As both methods are non-toxic and non-invasive, they can be applied sequentially to the same samples over time without compromising sterility. While the CCK-8 assay offers a quantitative assessment of cell proliferation, ultrasonic methods exhibit superior sensitivity to alterations in matrix microarchitecture and viscoelastic properties, including the deposition of native ECM. The synergy of these approaches enables a reduction in the required number of experimental samples, enhances research accuracy, and facilitates the effective screening of TECs for subsequent implantation via rapid testing. Collectively, these results represent a significant step toward developing a reliable in vitro control system for platforms used in the study of tissue pathology and regenerative medicine strategies.

## Figures and Tables

**Table 1:** Molecular weight of the samples.

Sample	Mw Intact, kDa (PDI)	Mw 0 Weeks in Medium, kDa (PDI)	Mw 4 Weeks in Medium, kDa (PDI)
PLLA	102 (2.3)	95 (2.0)	90 (2.0)
chitosan-PLLA	97 (2.0)	94 (2.1)	90 (2.1)

**Table 2:** Degree of crystallinity for studying samples.

Sample	Intact, %	0 Weeks in Medium, %	4 Weeks in Medium, %
PLLA	0	0	10
chitosan-PLLA	0	0	10

**Table 3:** Linear approximation slope of frequency dependence $R_{S}^{\prime }\left(f\right)$, dB/mm/MHz.

Sample	Precondition	3 Day TECs	10 Day TECs	17 Day TECs
PLLA	0.18	0.13	0.14	0.19
chitosan-PLLA	0.13	0.13	0.16	0.14

**Table 4:** Dynamic changes in the key components of the TECs during in vitro cultivation.

Sample	Component	Stage				
		Dry Matrix	Precondition Matrix	3 Days TECs	10 Days TECs	17 Days TECs
**PLLA**	**fibers**	unchanged state	unchanged state	smoothed surface by cells	smoothed surface by cells	smoothed surface by cells
	**cells**	no	no	appearance	proliferation	proliferation
	**ECM**	no	no	no	appearance	evolution *
**Chitosan-PLLA**	**fibers**	partially bonded with chitosan	smoothed surface by swollen chitosan	smoothed surface by swollen chitosan and cells	smoothed surface by swollen chitosan and cells	smoothed surface by swollen chitosan and cells
	**chitosan**	dry films	swollen	swollen	swollen, start degradation	swollen, degradation
	**cells**	no	no	appearance	lower proliferation	lower proliferation
	**ECM**	no	no	no	appearance	remodeling *

* requires following validation with histochemical methods; evolution means systematic step-by-step development; remodeling means nonlinear unpredictable behavior.

## Data Availability

The original contributions presented in this study are included in the article. Further inquiries can be directed to the corresponding author.

## Abbreviations

The following abbreviations are used in this manuscript:

AoS	attenuation of sound
chitosan-PLLA	PLLA non-woven matrix modified with chitosan
DMEM	Dulbecco’s Modified Eagle Medium
DSC	differential scanning calorimetry
ECM	extracellular matrix
GPC	gel permeation chromatography
PBS	phosphate-buffered saline
PLLA	PLLA non-woven matrix
SBW	spectrum bandwidth
SIAM	scanning impulse acoustic microscopy
SoS	speed of sound
TEC	tissue engineering construct

## References

[1] Baker B.M., Chen C.S. (2012). Deconstructing the third dimension: How 3D culture microenvironments alter cellular cues. J. Cell Sci..

[2] D’Urso M., Kurniawan N.A. (2020). Mechanical and Physical Regulation of Fibroblast-Myofibroblast Transition: From Cellular Mechanoresponse to Tissue Pathology. Front. Bioeng. Biotechnol..

[3] Romo-Uribe A., Meneses-Acosta A., Domínguez-Díaz M. (2017). Viability of HEK 293 cells on poly-β-hydroxybutyrate (PHB) biosynthesized from a mutant Azotobacter vinelandii strain. Cast film and electrospun scaffolds. Mater. Sci. Eng. C.

[4] Kennedy K.M., Bhaw-Luximon A., Jhurry D. (2017). Cell-matrix mechanical interaction in electrospun polymeric scaffolds for tissue engineering: Implications for scaffold design and performance. Acta Biomater..

[5] Trofimchuk E.S., Potseleev V.V., Khavpachev M.A., Moskvina M.A., Nikonorova N.I. (2021). Polylactide-Based Porous Materials: Synthesis, Hydrolytic Degradation Features, and Application Areas. Polym. Sci. Ser. C.

[6] Bianchi E., Ruggeri M., Vigani B., Aguzzi C., Rossi S., Sandri G. (2024). Synthesis and use of thermoplastic polymers for tissue engineering purposes. Int. J. Pharm. X.

[7] Malikmammadov E., Tanir T.E., Kiziltay A., Hasirci V., Hasirci N. (2018). PCL and PCL-based materials in biomedical applications. J. Biomater. Sci. Polym. Ed..

[8] Tenchurin T.K., Rodina A.V., Saprykin V.P., Gorshkova L.V., Mikhutkin A.A., Kamyshinsky R.A., Yakovlev D.S., Vasiliev A.L., Chvalun S.N., Grigoriev T.E. (2022). The Performance of Nonwoven PLLA Scaffolds of Different Thickness for Stem Cells Seeding and Implantation. Polymers.

[9] Chen F.M., Liu X. (2016). Advancing biomaterials of human origin for tissue engineering. Prog. Polym. Sci..

[10] Zamani R., Aval S.F., Pilehvar-Soltanahmadi Y., Nejati-Koshki K., Zarghami N. (2018). Recent Advances in Cell Electrospining of Natural and Synthetic Nanofibers for Regenerative Medicine. Drug Res..

[11] Muniyandi P., Palaninathan V., Veeranarayanan S., Ukai T., Maekawa T., Hanajiri T., Mohamed M.S. (2020). ECM Mimetic Electrospun Porous Poly (L-lactic acid) (PLLA) Scaffolds as Potential Substrates for Cardiac Tissue Engineering. Polymers.

[12] Perez-Puyana V., Wieringa P., Yuste Y., de la Portilla F., Guererro A., Romero A., Moroni L. (2021). Fabrication of hybrid scaffolds obtained from combinations of PCL with gelatin or collagen via electrospinning for skeletal muscle tissue engineering. J. Biomed. Mater. Res. A.

[13] Furuike T., Nagahama H., Chaochai T., Tamura H. (2015). Preparation and Characterization of Chitosan-Coated Poly(l-Lactic Acid) Fibers and Their Braided Rope. Fibers.

[14] Abdullah M.F., Nuge T., Andriyana A., Ang B.C., Muhamad F. (2019). Core–Shell Fibers: Design, Roles, and Controllable Release Strategies in Tissue Engineering and Drug Delivery. Polymers.

[15] Romanova O.A., Grigor’ev T.E., Goncharov M.E., Rudyak S.G., Solov’yova E.V., Krasheninnikov S.T., Saprykin V.P., Sytina E.V., Chvalun S.N., Pal’tsev M.A. (2015). Chitosan as a Modifying Component of Artificial Scaffold for Human Skin Tissue Engineering. Bull. Exp. Biol. Med..

[16] Kamyshinsky R.A., Patsaev T.D., Tenchurin T.K., Zagoskin Y.D., Grigoriev T.E., Darienko K.A., Panteleyev A.A., Chvalun S.N., Vasiliev A.L. (2020). Environmental scanning electron microscopy of dermal fibroblasts on different types of polymeric scaffolds. Crystallogr. Rep..

[17] Burkhardt C., Nisch W. (2005). Electron Microscopy on FIB prepared interfaces of biological and technical materials: First results. Pract. Metallogr..

[18] Drobne D., Sousa A., Kruhlak M. (2013). 3D Imaging of Cells and Tissues by Focused Ion Beam/Scanning Electron Microscopy (FIB/SEM). Nanoimaging: Methods in Molecular Biology.

[19] Tkachev S., Chepelova N., Galechyan G., Ershov B., Golub D., Popova E., Antoshin A., Giliazova A., Voloshin S., Efremov Y. (2024). Three-Dimensional Cell Culture Micro-CT Visualization within Collagen Scaffolds in an Aqueous Environment. Cells.

[20] Winterroth F., Lee J., Kuo S., Fowlkes J.B., Feinberg S.E., Hollister S.J., Hollman K.W. (2011). Acoustic microscopy analyses to determine good vs. failed tissue engineered oral mucosa under normal or thermally stressed culture conditions. Ann. Biomed. Eng..

[21] Ruland A., Gilmore K.J., Daikuara L.Y., Fay C.D., Yue Z., Wallace G.G. (2019). Quantitative ultrasound imaging of cell-laden hydrogels and printed constructs. Acta Biomater..

[22] Khramtsova E., Morokov E., Antipova C., Krasheninnikov S., Lukanina K., Grigoriev T. (2022). How the Nonwoven Polymer Volume Microstructure Is Transformed under Tension in an Aqueous Environment. Polymers.

[23] Lukanina K.I., Grigoriev T.E., Krasheninnikov S.V., Mamagulashvilli V.G., Kamyshinsky R.A., Chvalun S.N. (2018). Multi-hierarchical tissue-engineering ECM-like scaffolds based on cellulose acetate with collagen and chitosan fillers. Carbohydr Polym..

[24] Lionetto F., Maffezzoli A. (2009). Polymer characterization by ultrasonic wave propagation. Adv. Polym. Technol..

[25] Mori H., Norisuye T., Nakanishi H., Tran-Cong-Miyata Q. (2018). Ultrasound attenuation and phase velocity of micrometer-sized particle suspensions with viscous and thermal losses. Ultrasonics.

[26] Tsuji K., Nakanishi H., Norisuye T. (2021). Viscoelastic ECAH: Scattering analysis of spherical particles in suspension with viscoelasticity. Ultrasonics.

[27] Rodina A.V., Tenchurin T.K., Saprykin V.P., Shepelev A.D., Mamagulashvili V.G., Grigor’ev T.E., Moskaleva E.Y., Chvalun S.N., Severin S.E. (2017). Proliferative and differentiation potential of multipotent mesenchymal stem cells cultured on biocompatible polymer scaffolds with various physicochemical characteristics. Bull. Exp. Biol. Med..

[28] Cukierman E., Pankov R., Stevens D.R., Yamada K.M. (2001). Taking cell-matrix adhesions to the third dimension. Science.

[29] Antipova C.G., Lukanina K.I., Krasheninnikov S.V., Malakhov S.N., Kamyshinsky R.A., Grigoriev T.E., Chvalun S.N. (2021). Study of highly porous poly-L-lactide based composites with chitosan and collagen. PAT.

[30] Farrugia B.L., Brown T.D., Upton Z., Hutmacher D.W., Dalton P.D., Dargaville T.R. (2013). Dermal fibroblast infiltration of poly(ε-caprolactone) scaffolds fabricated by melt electrospinning in a direct writing mode. Biofabrication.

[31] Fischer E.W., Sterzel H.J., Wegner G. (1973). Investigation of the structure of solution grown crystals of lactide copolymers by means of chemical reactions. Kolloid-Z. Z. Polym..

[32] Kulikova O., Khramtsova E., Lukanina K., Patsaev T., Morokov E., Antipova C., Levin V., Grigoriev T. (2021). Ultrasonic assessment of the distribution of tricalcium phosphate filler over the volume of swollen porous matrices based on chitosan for biomedical applications. J. Biomed..

[33] Lizzi F.L., Greenebaum M., Feleppa E.J., Elbaum M., Coleman D.J. (1983). Theoretical framework for spectrum analysis in ultrasonic tissue characterization. J. Acoust. Soc. Am..

[34] Bereiter-Hahn J., Salzer R. (2012). Acoustic Microscopy for Biomedical Applications. Biomedical Imaging: Principles and Applications.

[35] Sorriento A., Poliziani A., Cafarelli A., Valenza G., Ricotti L. (2021). A novel quantitative and reference-free ultrasound analysis to discriminate different concentrations of bone mineral content. Sci. Rep..

[36] Taggart L.R., Baddour R.E., Giles A., Czarnota G.J., Kolios M.C. (2007). Ultrasonic characterization of whole cells and isolated nuclei. Ultrasound Med. Biol..

[37] Wear K.A., Member S. (2020). Mechanisms of interaction of ultrasound with cancellous bone: A review. IEEE Trans. Ultrason. Ferroelectr. Freq. Control..

[38] Bauer A.Q., Anderson C.C., Holland M.R., Miller J.G. (2009). Bone sonometry: Reducing phase aberration to improve estimates of broadband ultrasonic attenuation. J. Acoust. Soc. Amer..

[39] Xu W., Kaufman J.J. (1993). Diffraction correction methods for insertion ultrasound attenuation estimation. IEEE Trans. Biomed. Eng..

[40] Omari E., Lee H., Varghese T. (2011). Theoretical and phantom based investigation of the impact of sound speed and backscatter variations on attenuation slope estimation. Ultrasonics.

